# CLAAF: Multimodal fake information detection based on contrastive learning and adaptive Agg-modality fusion

**DOI:** 10.1371/journal.pone.0322556

**Published:** 2025-05-07

**Authors:** Guangyu Mu, Chuanzhi Chen, Xiurong Li, Ying Chen, Jiaxiu Dai, Jiaxue Li

**Affiliations:** 1 School of Management Science and Information Engineering, Jilin University of Finance and Economics, Changchun, China; 2 Key Laboratory of Financial Technology of Jilin Province, Changchun, China; 3 Faculty of Information Technology, Beijing University of Technology, Beijing, China; Public Library of Science, UNITED KINGDOM OF GREAT BRITAIN AND NORTHERN IRELAND

## Abstract

The widespread disinformation on social media platforms has created significant challenges in verifying the authenticity of content, especially in multimodal contexts. However, simple modality fusion can introduce much noise due to the differences in feature representations among various modalities, ultimately impacting the accuracy of detection results. Thus, this paper proposes the Contrastive Learning and Adaptive Agg-modality Fusion (CLAAF) model for multimodal fake information detection. Firstly, a contrastive learning strategy is designed to align text and image modalities, preserving essential features while minimizing redundant noise. Secondly, an adaptive agg-modality fusion module is proposed to facilitate deep interaction and integration between modalities, enhancing the model’s capability to process complex multimodal information. Finally, a comprehensive multimodal dataset is constructed through web crawling from authoritative news sources and multiple fact-checking platforms, establishing a solid foundation for training and validating the model. The experimental results demonstrate that the CLAAF model achieves a 3.45% improvement in accuracy compared to the best-performing baseline models, observably advancing the precision and robustness of multimodal fake information detection.

## Introduction

According to a report from the Pew Research Center in 2021, 86% of American adults obtain information through social media platforms [1]. While platforms like Facebook, Instagram, and Sina Weibo enhance the convenience of accessing information, they have also inadvertently accelerated the spread of fake information [2,3]. Fake information refers to unverified content. It plays a vital role in its generation, dissemination, and impact on online platforms [4]. Since 2020, Facebook and Instagram have deleted more than 20 million instances of fake information [5], while Sina Weibo has dealt with 66,251 instances of fake information in 2021 [6]. The rapid and widespread dissemination of fake information through various channels makes timely verification challenging, resulting in numerous negative societal impacts. Thus, it is critical to address the detection of fake information promptly.

In the fight against misinformation and to counter misleading or inaccurate content, there have been notable advancements in independent verification services and content regulation tools [7–13]. When content detection techniques evolve, fake information has shifted from unimodal text-based formats to multimodal formats that combine text and images. Compared to traditional text-based content, multimodal information that integrates text and images can more effectively convey event details and capture the reader’s attention. Furthermore, fake information that blends actual events with fabricated content is more deceptive and likely to spread widely, posing a great challenge for online platforms to regulate validly. As a result, researchers are increasingly interested in utilizing advanced technology to detect multimodal fake information.

The development of multimodal fake information detection systems has progressed from analyzing unimodal to multimodal content to determining content authenticity. Early approaches focus on directly integrating representations from various modalities to detect fake information. For instance, some scholars proposed a multimodal model named SpotFake, which could learn contextual information from input textual and visual data by BERT and VGG19 [14]. Attention-based methods [15] detect fake online information by identifying dependencies and connections between text and images through the cross-attention mechanism. Although these methods have been practical, combining image and text features yields may cause inconsistent results. The accuracy of information depends not only on the correlation but also on the alignment of meaning between textual and visual elements. Low semantic similarity can introduce noise. Attention-based methods are vulnerable to input interference, where even small perturbations can alter attention weights and impair the fusion mechanism.

Due to the limitations of attention-based methods, many researchers have adopted the approach of concatenating feature vectors. However, this method presents another challenge in differentiating low-level visual characteristics in images from high-level semantic notions in text. Specifically, this approach treats each modality as an independent information source and merges unimodal feature vectors into one unified vector for categorization. The concatenation fusion does not adequately capture the nuanced concern between different modalities and ensure the proper alignment of modal information, potentially introducing noise [16]. Therefore, better alignment of unimodal information and more effective multimodal fusion are crucial for improving multimodal fake information detection.

This paper proposes a model for detecting fake information called Contrastive Learning and Adaptive Agg-modality Fusion (CLAAF). The model comprises two core modules: a modality alignment module and an adaptive agg-modality fusion module. The modality alignment module aims to effectively align and preserve key features while discarding redundant information, enhancing the consistency and quality of feature representations across modalities. The adaptive agg-modality fusion module learns and integrates multimodal information through layer-by-layer aggregation and alignment of features. In this process, text features drive the optimization of image feature representations, creating stronger inter-modal connections and improving the precision of detecting false information. The efficiency and advantages of the proposed model in social media fake information detection are validated using a self-scraped dataset.

The main contributions of this paper are as follows:

1) A novel module aligns modalities using contrastive learning, enhancing feature consistency and quality by discarding redundant information.
2) An adaptive aggregation-modality fusion module is proposed to establish closer inter-modal connections and enhance the accuracy of detecting fake information.
3) An expanded, genuine, and varied multimodal dataset is constructed and validated, addressing the limitations of existing datasets in terms of scale and diversity.

The rest of this paper is structured as follows: Section 2: Related work reviews existing methods for fake information detection. Section 3: Methodology provides an overview of the proposed framework, including modality encoding, modality alignment, and multimodal fusion. The experimental setup, performance comparisons, and results evaluation are presented in Section 4: Experiments analysis. Section 5: Conclusion and future work discuss the conclusion.

## Related work

Identifying false information is typically classified into two types depending on the modality: unimodal and multimodal detection approaches.

### Unimodal fake information detection

Unimodal fake information detection initially relies on manually extracted features, such as statistical analysis of punctuation marks, memorable characters, and word frequency distributions [17]. However, manual feature extraction is complex, time-consuming, and unsuitable for large-scale data processing. With the advent of deep learning, research has focused more on using deep learning models for automatic feature extraction. Typical deep learning architectures include CNNs [18], RNNs [19], and their variations like LSTM [20] and GRU [21], as well as models incorporating attention mechanisms. For instance, CNN-based models can uncover deep textual features through the learning process in hidden layers. RNN excel at capturing latent spatiotemporal semantic features, performing well on specific datasets [22,23]. Some models achieve superior detection performance by combining CNN and GRU to extract deep textual features and temporal sequence information [24]. Another approach integrates tree structures with RNN to analyze textual content and user comments to yield promising results. However, this method depends on commenting and reposting information, which introduces a certain degree of delay [25].

As pre-trained models like BERT evolve, scholars have begun exploring using BERT for vectorized text representation. They combine it with other neural network models for fake information detection and achieve significant results [26]. Further studies have investigated modified hybrid neural networks based on the BERT model. BERT’s vectorized representations are integrated with Bi-LSTM and attention mechanisms to enhance detection performance [27].

However, these methods either incur high computational costs or utilize only textual information, limiting the upper bound of model detection accuracy.

### Multimodal fake information detection

#### Detection based on multimodal features.

Early methods for multimodal misinformation detection focus on combining features from different modalities to enhance detection accuracy. These approaches leverage the complementarity of multimodal data to extract more diverse and resilient features [28–32]. For example, the EANN model combines Text-CNN with VGG-19 to strengthen the integration of modalities [28]. Additionally, variational encoders were employed to capture probabilistic latent variables, enhancing cross-modal associations [29]. CLIP-guided learning uses the CLIP model to guide the alignment of text and image modalities, significantly enhancing the consistency of cross-modal features [31]. Some scholars have also proposed multi-view representation learning, which further enhances the model’s ability to identify complex misinformation through augmented processing of multimodal data [32]. The above methods highlight the advantages of fusing multimodal information to detect fake information. Some extended models incorporate information from various abstraction levels using text, images, user feedback, and metadata. At the same time, these models maintain the intrinsic structure of each modality through a staged fusion strategy [30]. Moreover, a multimodal deep learning model has been developed to combine text, image, and social attributes. However, this method does not consider the similar features of images [33].

These studies indicate that integrating multimodal features can enhance the model’s performance. Nevertheless, merely merging visual and textual features does not consistently guarantee accurate information extraction. Due to potential inconsistency between text and images, directly fusing features without proper alignment often leads to information distortion, resulting in feature redundancy, poor fusion performance, and challenges in model training.

#### Detection based on multimodal consistency.

The inconsistency between text and images is a significant problem in multimodal fake information detection. The inconsistency happens when the text and images do not match or contradict each other. For example, a natural disaster news is paired with a beautiful landscape photo, which can mislead the reader’s feelings. Besides, a description of a social event with a completely unrelated image weakens the image’s semantic complement to the text. The inconsistency increases the difficulty for the model to fuse and align features from multimodal data. Thus, the model must sort out valuable details from misleading or conflicting content.

In order to address this challenge, researchers have recently focused on mitigating the influence of deceptive content by evaluating the consistency between textual and visual components [34–37]. Some studies have introduced similarity measurement techniques to assess the coherence of multimodal data [38]. For example, the MCNN model maps multimodal features into a shared space and assesses their correlation by calculating their cosine similarity [39]. TRIMOON model significantly improves the classification performance on Chinese and English datasets by conducting two rounds of inconsistency detection and multimodal feature [35].

Despite the successes of previous methods, model performance may still suffer if feature alignment needs to be improved or irrelevant features are retained during extraction. In recent years, scholars have further explored how to improve the effect of consistency detection by improving feature alignment techniques. Some studies employ self-attention techniques to enhance the semantic alignment between different modalities using the self-attention mechanism to reduce the interference of irrelevant features [40]. In addition, some studies introduce adversarial learning mechanisms to create visual features that align closely with the text. This process improves the robustness of the model in multimodal consistency detection [41].

Although the current research has made some progress in multimodal consistency detection, many limitations remain. On the one hand, multimodal consistency detection highly depends on accurate feature alignment, which often requires complex model structures and enormous computational resources. On the other hand, the noise and heterogeneity of data from different modalities will also harm the effect of consistency detection.

### Multimodal fusion

Multimodal fusion is critical in fake information detection, as it integrates features from various modalities to enhance model performance. The majority of feature fusion algorithms rely on concatenation operation. Text and visual modality features are merged into one vector and then transferred to the neural network to proceed to the next steps [42]. Nevertheless, this method needs help to fully capture the intricate connections between textual and visual modalities. The method treats them as independent information sources and overlooks their potential interactions. Early studies sought to concatenate features derived from various techniques and sources without considering the interactions between these features [43]. Subsequent research introduced attention mechanisms, which enhanced fusion techniques by enabling the model to concentrate on interactions between different types of features and user-related information. However, these methods primarily depend on the cooperation of local features and need to fully leverage the potential of global feature interactions [44,45].

As research progresses, more approaches focus on deep learning strategies that capture multimodal features through various modules [38,39,46–48]. An approach is introduced to improve the alignment between text and image features via a fusion module, incorporating a loss function that measures semantic distance [38]. Some researchers utilize cross-attention mechanisms and complex networks to improve modal feature correlations. However, they must fully consider the fine-grained relationships within individual modalities [46]. Some scholars introduce a multimodal fusion model based on attention mechanisms and Bayesian optimization to enhance the correlation between diverse features [47]. In addition, some researchers develop multimodal frameworks capable of generating holistic representations of social media posts [39] or propose collaborative learning methods that combine relational representation graphs with a layered attention mechanism to facilitate communication between network nodes [48].

Even though these methods have made significant strides, they still have flaws. Simply concatenating can introduce irrelevant information, leading to more noise in the detection algorithm. Moreover, using different modules to handle multimodal features can make the model more prone to noise during extraction, potentially affecting its ability to identify nuanced relationships within each data type.

## Methodology

This section describes the overall model architecture, including task definition, modality encoding, modality alignment, multimodal fusion, and the loss function.

### Define task

This study’s multimodal fake information detection primarily involves text t and visual v. The primary goal is to align modalities and facilitate efficient interactive fusion, combining intra-modal and inter-modal features to detect fake information accurately. The task is defined as follows: Given a dataset D consisting of N sequence data points {$x_{1}$, $x_{2}$, $x_{3}$, ⋯ , $x_{n}$} and their corresponding labels {$y_{1}$, $y_{2}$, $y_{3}$, ⋯ , $y_{n}$}, each sequence data point $x_{i}\in \;R^{l_{m}\times d_{m}}$ includes associated visual features $v_{i}$ and textual features $t_{i}$. Here, $x_{i}\in \;\{v_{i},\;t_{i}\}$, where $l_{m}$ represents the sequence length, and $d_{m}$ denotes the vector dimension of modality m. The task of multimodal fake information detection is to assign a category $y_{i}$ to each input $x_{i}$, where$y_{i}$ indicates whether the information is fake or not.

### Create modality encoding

First, the input $X_{m}$ of the multimodal sequence is encoded, and two Transformer layers are introduced for each modality to unify feature representation. Specifically, a low-dimensional token matrix $H_{m}^{0}\in R^{T\times d_{m}}$ is randomly initialized for each modality. The basic modality information is then embedded into these tokens using the Transformer.

$H_{m}^{1}\;=\;Transformer(Concat(H_{m}^{0},\;X_{m}),\theta_{m})\in R^{T\times d}$ (1)

Here, $H_{m}^{1}$ represents the unimodal features for each modality, $\theta_{m}$ denotes the parameters of the current Transformer, and Concat(·) signifies the concatenation operation, with T as the length and d as the dimension. The T tokens can condense and integrate different unimodal features through the self-attention mechanism. This operation shares only the essential information within each modality, enhancing multimodal fusion performance while reducing computational complexity. In the experiments, T and d are set to 8 and 128, respectively, and the depth of the Transformer layer is set to 1. Transferring the basic modality information to the initialized low-dimensional tokens helps reduce redundant information, achieving higher efficiency with fewer parameters.

### Design modality alignment

Contrastive Predictive Coding (CPC) is a self-supervised learning method that enables the model to learn the intrinsic structure of data unsupervised by introducing contrastive learning. In this study, CPC is used to align the features of text and image modalities, achieving consistency between the two modalities in a shared feature space. Specifically, CPC compresses high-dimensional data into a more compact latent space to reduce redundant information. At the same time, CPC uses an autoregressive model to predict multiple future steps and simplifies the conditional prediction modeling process. Thus, CPC could help the model optimize the modality alignment effect and enhance the feature representation.

Noise-Contrastive Estimation (NCE) is a core mechanism of CPC contrastive learning. It improves the model by comparing positive and negative samples, which allows the entire model to be optimized end-to-end. Using many negative samples, NCE helps the model clearly identify the pairs of features most important to the anchor samples. In CPC, the model needs to discern the positive sample relationships between the text modality and its corresponding visual modality and ignore irrelevant negative samples. The quality of feature representation is significantly improved and the model’s discriminative ability in the feature space is enhanced by this design.

The specific calculation steps for CPC loss are as follows. First, each modality feature is normalized to align the scales of different modalities in the feature space. The text features are defined as ${\hat{X}}_{o}$, and the visual modality features are $h_{v}$. The normalized feature representation is shown in Equation 2–3. Here, $G_{\emptyset }$ represents a neural network parameterized by ∅, which calculates the distance between the fused features.

$\overset{―}{G_{\emptyset }\left({\hat{X}}_{o}\right)}\;=\;\frac{G_{z}\left({\hat{X}}_{o}\right)}{{\left\|G_{z}\left({\hat{X}}_{o}\right)\right\|}_{2}}$ (2)

$\overset{―}{h_{v}}\;=\;\frac{h_{v}}{{\left\|h_{v}\right\|}_{2}}$ (3)

Then, the similarity scoring function is used to incorporate other modality representations from the same batch as negative samples into the noise contrastive similarity estimation. As Equations 4 shows.

$s\left(h_{v},{\hat{X}}_{o}\;\right)\;=\;\exp\left(\overset{―}{h_{v}}{\left(\overset{―}{G_{z}\left({\hat{X}}_{o}\right)}\right)}^{T}\right)$ (4)

Additionally, by adjusting the contribution ratio of each modality, the model’s predictions determine the amount of information to be extracted from each modality, as detailed in Equations 5 and 6.

${ℒ}_{N}^{{\hat{X}}_{o},h_{v}\;}\;=\;-\text{E}\left[\log\frac{s\left(h_{v}^{i},{\hat{X}}_{o}\;\right)}{\sum s(h_{v}^{j},{\hat{X}}_{o}\;)}\right]$(5)

${ℒ}_{CPC}\;=\;{ℒ}_{N}^{{\hat{X}}_{o},h_{v}\;}\;$ (6)

In the experiment, each text anchor sample is paired with two positive samples and 2,000 negative samples. CPC constructs an optimization problem through this positive-negative sample comparison method, and helps the model to accurately identify positive samples among a large number of negative samples. In Equation 5, the numerator $s\left(h_{v}^{j},{\hat{X}}_{o}\;\right)$ represents the similarity of positive sample pairs, while the denominator contains the sum of similarities of all positive and negative samples. The model achieves its goal by maximizing the ratio of the numerator to the denominator. In this way, it maximizes the similarity of positive sample pairs and minimizes the similarity of negative sample pairs. In the end, positive sample pairs are gathered in the shared feature space, and negative sample pairs are effectively dispersed. Through contrastive learning, this approach not only achieves alignment between text and image modality features but also discards redundant features in the text during the optimization process, thereby enhancing the expression of critical features. This method effectively improves the modality alignment, resulting in more consistent representations of text and images in the feature space.

### Propose a novel multimodal fusion

Detecting multimodal disinformation aims to align the features of two modalities, maximize their interactive information, and guide the learning of unimodal features. After aligning the modalities, an adaptive agg-modality fusion module with aggregation blocks is designed to learn refined multimodal representations. This module incorporates modality-consistent and modality-specific information, using text features as the guide while enhancing the text features. The module consists of two transformer layers and multiple cross-modal aggregation layers. It is introduced to learn text features at different scales and adaptively learn image modality features under the guidance of the text features.

#### Define text multi-scale features.

Let $H_{t}^{1}$ represent the low-scale text features, and two transformer layers are introduced to learn the mid-scale features $H_{t}^{2}$ and high-scale features $H_{t}^{3}$. The Transformer layers at this stage directly model the linguistic features, as shown in Equation 7.

$H_{t}^{i}\;=\;Transformer(H_{t}^{i-1},\;\theta_{t}^{i})\in R^{T\times d},\;i\in \left\{2,\;3\right\}$ (7)

Here, $H_{t}^{i}$ represents the text features at different scales, and $\theta_{t}^{i}$ denotes the parameters related to the i-th layer.

#### Formulate an adaptive agg-modality module.

In traditional Cross-Attention mechanisms, a modality must be updated twice during interaction to enhance modality. This process leads to inefficiencies and the generation of redundant features. A global-local learning model with linear computational cost is designed to address this issue and improve parameter efficiency. The discourse level representation of each modality can substitute standard information and interact with the local unimodal features as the global multimodal context $H_{agg}^{0}$. The text features continue to play a guiding role in the above process.

The specific operation is to set the global multimodal context information $H_{agg}^{0}\in R^{T\times d}$. The model learns both modality consistency and specificity by interacting global information with local modality information. The multimodal aggregation module $H_{agg}^{0}$ is initialized using the multi-scale language features $H_{t}^{i}$. Then, the relationship between text and image modalities is updated through multi-head attention. For example, a similarity matrix α between text features and image features can be obtained using the extracted $H_{t}^{i}$ as the query and $H_{v}^{1}$ as the key.

$\alpha \;=\;softmax(\frac{Q_{t}K_{v}^{T}}{\sqrt{d_{k}}}=\;softmax(\frac{H_{t}^{i}W_{Q_{t}}W_{K_{v}}^{T}H_{v}^{1T}}{\sqrt{d_{k}}})\;\in R^{T\times T}$ (8)

As shown in Equation 8, the softmax operation represents the weight normalization, where $W_{Q_{t}}\in \;R^{d\times d_{k}}$ and $W_{K_{v}}\in \;R^{d\times d_{k}}$ are learnable parameters, and $d_{k}$ is the dimension of each attention head. Similarly, the self-attention similarity matrix for image features β can be obtained, as shown in Equation 9.

$\beta \;=\;softmax(\frac{Q_{v}K_{v}^{T}}{\sqrt{d_{k}}}=\;softmax(\frac{H_{v}^{i}W_{Q_{v}}W_{K_{v}}^{T}H_{v}^{1T}}{\sqrt{d_{k}}})\;\in R^{T\times T}$ (9)

Here, $W_{K_{v}}\in \;R^{d\times d_{k}}$ is a learnable parameter. The image features start receiving triple guidance from $H_{agg}^{1}$: text information, image information, and the aggregated information from the previous layer. The features are updated by weighting the cross-modal features with the self-attention features, as shown in Equation 10.

$H_{agg}^{j}\;=\;H_{agg}^{j-1}\;+\;\alpha H_{v}^{1}W_{V_{v1}}\;+\;\beta H_{v}^{1}W_{V_{v2}},\;j\in \left\{1,\;2,\;3\right\}$ (10)

Here, $H_{agg}^{j}$ represents the aggregated features at the j-th layer, while $W_{V_{v1}}$ and $W_{V_{v2}}$ are learnable parameters designed to capture complex global-local interactions across different modules. As layers accumulate, the whole multimodal context and partial unimodal features undergo continuous refinement and reciprocal reinforcement.

#### Time complexity analysis.

The original time complexity is $O\left(\sum_{m=1}^{M}{T_{m}}^{2}\right)$, where M is the number of modalities and $T_{m}$ is the length of each modality. The number of modalities M is typically small, such as the two modalities used in this study. However, the length $T_{m}$ of each modality can be pretty big. Text modalities may consist of hundreds to thousands of words, and image modalities may have dimensions of hundreds to thousands of pixels. Given that $M\;\ll \;T_{m}$, the time complexity can be simplified to $O\left(MT^{2}\right)$.

Therefore, the designed global-local fusion strategy exhibits linear space complexity and benefits from linear computational efficiency in modality processing. The model initially learns shallow interactive features and subsequently focuses on high-level semantic features. The model integrates information from different modalities by designing aggregation blocks and provides a more comprehensive and rich multimodal feature representation. Enabling deeper interaction between modalities allows the model to extract more advanced and abstract feature representations in the later stages of learning.

### Output multimodal fusion

Finally, the aggregated features $H_{agg}^{3}$ and the text features $H_{t}^{3}$ undergo cross-modal attention computation to obtain the final output class $\hat{y}$ for fake information detection, as shown in Equation 11.

$\hat{y}\;=\;CrossTransformer(H_{t}^{3},\;H_{agg}^{3})$ (11)

**Fig 1:**
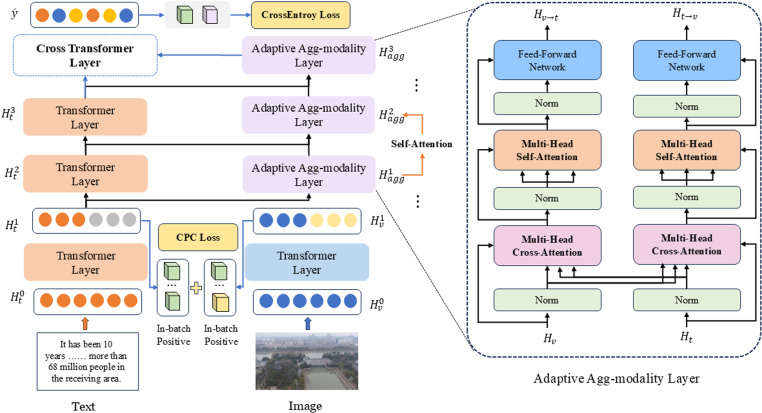
Multimodal Fake Information Detection Model CLAAF.

In summary, the novel multimodal fake information detection model constructed in this study is illustrated in Fig 1.

### Loss function

In addition to the CPC loss, the model incorporates a cross-entropy loss function and a total loss function specifically for the detection task. In multimodal fake information detection, the cross-entropy loss is commonly used. The goal is to reduce the gap between predicted and actual data distributions, improving the accuracy of fake information detection. Therefore, the cross-entropy loss is employed for the classification task, as defined in Equation 12.

${ℒ}_{pred}=\sum_{i\in \{t,v,f\}}^{N}\left[y^{i}\log{\hat{y}}^{i}+\left(1-y^{i}\right)\log\left(1-{\hat{y}}^{i}\right)\right]$ (12)

Here, ${ℒ}_{pred}$ is the classification loss function, $y^{i}$ represents the valid label of the sample (0 for non-fake information, 1 for fake information), ${\hat{y}}^{i}$ is the model’s prediction for the sample, and N is the total number of samples.

The total loss is obtained by summing the CPC loss with the classification loss, resulting in the final optimization objective, as shown in Equation 13, where α is a parameter that adjusts the weighting of the loss functions.

$L\;=\;{ℒ}_{pred}\;+\;{\alpha ℒ}_{CPC}\;$ (13)

## Experiments analysis

### Experimental parameters

Our experiments use an RTX 4090 GPU for training with an AdamW optimizer. The learning rate is set to 1e-4 and dynamically adjusted using a cosine annealing schedule. The specific parameter configurations are detailed in Table 1. The dataset is divided into 80% for training and 20% for testing.

**Table 1:** Experimental parameter setting.

Module Name	Parameter Values
T	8
d	128
α	0.1
Unimodal Depth	1
Multimodal Depth	3
Fusion Depth	2
Batch Size	64
Learning Rate	1e-4
Epochs	100

To assess the effectiveness of the proposed model, we choose accuracy, precision, recall, and F1-score as metrics. Table 2 uses TR and TF to denote actual real and fake, identifying the model and correctly predicting real and fake examples. Conversely, FR and FF represent false real and fake, indicating when the model incorrectly predicts real and fake examples. We provide the formulas for calculating these evaluation metrics in Equations 14–17.

**Table 2:** Confusion matrix of fake information detection.

Prediction Actual	Positive	Negative
Real	True Real (TR)	False Real (FR)
Fake	False Fake (FF)	True Fake (TF)

$Accuracy=\frac{TP+TN}{TP+FN+FP+TN}$ (14)

$Precision=\frac{TP}{TP+FP}$ (15)

$Recall=\frac{TP}{TP+FN}$ (16)

$F1-score=\frac{2*Precision*Recall}{Precision+Recall}$ (17)

### Data collection and preprocessing

This study collects data from CCTV.com and various fact-checking platforms using web scraping technology to validate the model’s effectiveness. The dataset comprises verified real information published on CCTV.com between April 2022 and November 2023. After removing irrelevant or meaningless content, we obtain 2817 pairs of text and image data. Fake information comes from platforms such as the China Internet Joint Rumor Debunking Platform, Science Rumor Debunking, Popular Science China, and Kedou Wuxianpu, covering debunked information from August 2019 to December 2023. After applying the same processing methods as the real information, 3,605 entries were retained.

The collection and use of the dataset strictly adhere to the terms and conditions of its source platform. All data comes from publicly available sources and does not involve sensitive or personal privacy information. The analysis and processing of data in this study are solely for academic research purposes. Additionally, no re-labeling or processing operations that could compromise data integrity are introduced during the research process. We ensure the legality of the data and the research’s compliance.

### Comparative experiments

The experiments include detecting fake information using unimodal methods, multimodal approaches driven by different fusion strategies, and comparisons with baseline models.

#### Comparative experiments for unimodal detection.

The purpose of the unimodal comparison experiment is to find the best-performing architecture. To achieve this goal, we compare unimodal classifiers based on different architectures for our model. This set of experiments includes multiple widely used high-performance unimodal classifiers, including TextCNN, BiLSTM, BERT, CLIP-Text, and RoBERTa for text, as well as CLIP-Image, ResNet, and ViT for image. The experiment uses the dataset we created, but the text and images are separated to target different classifiers. Table 3 shows experimental results.

**Table 3:** Unimodal detection results.

Method	Module	Accuracy	Precision	Recall	F1-score
TextCNN	T	0.8277	0.8194	0.8203	0.8204
Bi-LSTM	T	0.8304	0.8291	0.8298	0.8301
BERT	T	0.8695	0.8688	0.8685	0.8686
CLIP-Text	T	0.8725	0.8696	0.8699	0.8698
**RoBERTa**	**T**	**0.8873**	**0.8866**	**0.8861**	**0.8863**
CLIP-Image	V	0.8473	0.8451	0.8456	0.8455
ResNet	V	**0.8566**	0.8498	0.8495	0.8496
**ViT**	**V**	0.8542	**0.8515**	**0.8511**	**0.8513**

The results indicate that RoBERTa is the best method for text feature representation. Its accuracy is 7.20%, 6.85%, 2.05%, and 1.70% higher than that of TextCNN, Bi-LSTM, BERT, and CLIP-Text, respectively. The results indicate that the Transformer-based RoBERTa has a significant advantage in capturing text semantic features. Although ResNet’s accuracy is 0.28% higher than ViT regarding image feature extraction, it lags behind ViT in precision, recall, and F1-score. The comprehensive analysis suggests that ViT is the optimal method for image feature representation.

The above results show that Transformer-based models excel at complex classification tasks.

- Powerful global feature-capturing ability. The Transformer model uses self-attention to focus on feature interactions dynamically throughout the input sequence. This method effectively solves long-distance dependency problems in text, like understanding complex relationships in misinformation. The Transformer model can recognize overall spatial features in images, making it more adaptable than traditional CNN methods.
- Efficient multi-layer representation learning. The stacked structure of Transformers allows the model to extract different feature hierarchies at each layer. For example, RoBERTa can capture more abstract semantic representations at higher layers, while ViT can learn the balance between global semantics and local details of images at deeper layers.
- Flexible input handling function. Compared to traditional CNN models, ViT can handle image patches of non-fixed sizes, while RoBERTa demonstrates good adaptability to variable-length text sequences. This flexibility enhances the model’s applicability across diverse tasks.

The experimental results provide essential references for multimodal feature encoding strategies. Therefore, this paper designs a feature encoding module based on the Transformer architecture to enhance the expressive capability of modal features.

#### Comparative experiments for multimodal fusion methods.

This set of experiments is designed to assess the effectiveness of various fusion methods in multimodal fake information detection. Only the fusion module in the CLAAF model is replaced to ensure that the results are influenced solely by the fusion method, while other components remain unchanged. The validity and superiority of CLAAF are validated through comparisons with two widely used fusion methods named feature concatenation and attention-based fusion. Table 4 presents the experimental results.

**Table 4:** Fusion methods comparative results.

Method	Module	Accuracy	Precision	Recall	F1-score
Concatenation	T + V	0.9206	0.9199	0.9198	0.9198
Cross-Attention	T + V	0.9423	0.9419	0.9418	0.9418
**CLAAF(Ours)**	**T + V**	**0.9839**	**0.9838**	**0.9838**	**0.9839**

The experimental results make it clear that the adaptive agg-modality fusion module of CLAAF significantly outperforms the other two methods. The detailed analysis is as follows.

Concatenation (Feature Concatenation Fusion): This method directly fuses text and image features and feeds the vector into downstream tasks. Although the method is simple, it fails to fully exploit the complementary information between the two modalities, resulting in insufficient information fusion and the lowest performance among the three methods. In contrast, the fusion module of CLAAF improves accuracy by approximately 6.87%.

Cross-Attention (Attention-Based Fusion): This method extracts text and image interaction features through the cross-attention mechanism. Compared to concatenation, cross-attention better leverages complementary information between modalities, thereby enhancing the effectiveness of information fusion. However, it still lags behind CLAAF by about 4.41% in accuracy.

CLAAF (Proposed Method): The adaptive agg-modality fusion module combines text, image, and aggregated information from the previous layer through multi-level feature interaction. The fusion mechanism ensures that the features of each layer thoroughly interact and fuse. This approach effectively captures the complementary information between modalities. It visibly enhances the model’s robustness through multi-level feature fusion, resulting in superior performance across all four evaluation metrics compared to the other two methods.

### Comparative experiments for baseline models

To assess the proposed model, comparisons are made with six baseline models using the constructed dataset. Results are detailed in Table 5.

**Table 5:** Baseline model comparative results.

Method	Module	Accuracy	Precision	Recall	F1-score	Params	FLOPS
EANN [28]	T + V	0.8913	0.8707	0.8561	0.8616	334.46M	21.56G
QSAN [49]	T + V	0.9013	0.8873	0.8895	0.8884	791.37M	39.48G
SAFE [38]	T + V	0.9232	0.9087	0.9007	0.9051	954.65M	47.17G
MFAN [50]	T + V	0.9473	0.9131	0.9101	0.9124	1385.56M	59.28G
HSEN [51]	T + V	0.9485	0.9458	0.9449	0.9456	894.62M	45.88G
FSRU [52]	T + V	0.9511	0.9505	0.9512	0.9511	1204.36M	51.52G
**CLAAF(Ours)**	**T + V**	**0.9839**	**0.9838**	**0.9838**	**0.9839**	951.75M	48.54G

Results show that the CLAAF model surpasses all baseline models in every metric. Specifically, CLAAF improves accuracy by approximately 10.37% compared to EANN, 9.16% compared to QSAN, 6.58% compared to SAFE, 3.86% compared to MFAN, 3.73% compared to HSEN, and 3.45% compared to FSRU.

EANN [28]: This model enhances robustness through an event adversarial mechanism. While this approach somewhat improves the performance of multimodal fake information detection, its fusion, and utilization of multimodal features still need to be improved, preventing it from achieving optimal performance.

QSAN [49]: This model introduces a quantum self-attention mechanism and logic similarity, enhancing the model’s ability to process multimodal information through a quantized attention mechanism. However, despite its theoretical solid innovation, the complexity and computational overhead in practical applications are high, which prevents it from achieving optimal performance.

SAFE [26]: This model identifies fake news by detecting mismatches in cross-modal features, focusing on the relationship between text and images for fake information detection. Although this approach improves detection performance to some extent, it needs to understand the intricate interactions between features comprehensively.

MFAN [50]: This model integrates text, images, and social graph features within a unified framework, considering the complementary and alignment relationships between different modalities. MFAN significantly enhances the effectiveness of multimodal misinformation identification by inferring hidden links to optimize social graph features. However, the complex model structure and high computational cost still need to be addressed for MFAN.

HSEN [51]: This model employs reinforcement learning to generate image captions that match the text and uses an adaptive intricate attention mechanism to remove noise. While it improves the model’s robustness and accuracy, there is a need for improvement in global interaction and deep feature fusion of multimodal information, limiting its potential for further performance enhancement.

FSRU [52]: This model extracts highly discriminative spectral features using the Fourier transform and combines dual contrastive learning for multimodal representation and fusion. Although it excels in spectral feature extraction, its integration of non-spectral features and handling of complex multimodal interactions are limited, making its overall performance inferior to CLAAF.

CLAAF (Proposed Model): This model realizes the accurate alignment of text and image modalities through contrastive learning, reduces the noise and information redundancy between modalities, and enhances the consistency of feature expression. Additionally, the model utilizes an adaptive aggregation modality fusion module to integrate text and image features layer by layer. The approach fully utilizes the complementary relationship between modalities, significantly enhancing the efficiency of information fusion. The results indicate that the CLAAF model demonstrates excellent detection capabilities while maintaining low computational overhead.

#### Parameter analysis.

We use two metrics to indicate the detection efficiency of the model. The parameter (Params) refers to the total number of trainable parameters in the model. FLOPS refers to the number of floating-point operations a model can perform in one second. The lower these two metrics, the faster the model’s detection speed. Getting high detection performance while using fewer parameters and FLOPS shows that the model balances performance and efficiency well. This balance highlights its advantage in being efficient. Table 5 compares different models regarding the number of Params and FLOPS.

Although the CLAAF model has more parameters and FLOPS than the EANN model, the performance improvement is also substantial. Compared to other high-performance models like MFAN and FSRU, CLAAF achieves performance gains while maintaining a relatively reasonable computational complexity. Notably, among models with accuracy above 90%, the parameters and FLOPS of CLAAF are only slightly higher than a variant model where the CLAAF fusion method is replaced with Cross-Attention. Although this slightly increases the model’s complexity, it is justified given the significant performance improvement.

In summary, the CLAAF model balances performance and computational complexity well. Despite introducing complex adaptive agg-modality fusion modules and contrastive learning strategies, CLAAF maintains a relatively low level of parameters and computational complexity. Compared to other high-performance models, CLAAF improves detection effectiveness and demonstrates its efficiency and superiority in multimodal fake information detection tasks while maintaining reasonable computational costs.

### Ablation experiments

Ablation experiments are performed to evaluate how the alignment first and fusion later strategy influences the CLAAF model. The impact on overall performance is analyzed by gradually removing pivotal components of the model. Table 6 presents the experimental results.

**Table 6:** Ablation experiments results.

Method	Module	Accuracy	Precision	Recall	F1-score
w/o Agg	T+V	0.9603	0.9593	0.9586	0.9591
w/o CPC	T+V	0.9566	0.9512	0.9514	0.9512
w/o Agg, CPC	T+V	0.9423	0.9419	0.9418	0.9418
**CLAAF(Ours)**	**T+V**	**0.9839**	**0.9838**	**0.9838**	**0.9839**

w/o Agg: The adaptive agg-modality fusion module is removed to evaluate its influence on the model’s effectiveness.

w/o CPC: The contrastive learning strategy is removed, meaning no modality alignment is performed before fusion, to assess the influence of the contrastive learning strategy on the model’s performance.

w/o Agg, CPC: Both the adaptive agg-modality fusion module and the contrastive learning strategy are removed to evaluate the impact of removing both components on the model’s overall performance.

Ablation experiments show that the adaptive aggregation-modality fusion module and contrastive learning strategy in the CLAAF method significantly affect the model’s overall performance. The following is a detailed analysis of the results from each experiment.

w/o Agg: Accuracy decreases by 2.40%, precision by 2.49%, recall by 2.56%, and F1-score by 2.53%. The adaptive agg-modality fusion module enhances the model’s expressive power by capturing feature mutual information across different scales. After removing this module, the model cannot fully utilize the interaction information between multi-scale features, leading to a significant drop in performance. This process indicates that the module improves the model’s capability to interpret and handle intricate multimodal information.

w/o CPC: Accuracy decreases by 2.77%, precision by 3.31%, recall by 3.29%, and F1-score by 3.32%. The contrastive learning strategy enhances the consistency between modalities during alignment and improves the quality of feature representations. Using the contrastive learning strategy, the model can effectively align the features of different modalities before fusion. Removing this module leads to a decline in the effectiveness of information fusion and a significant impact on overall performance.

w/o Agg, CPC: Accuracy decreases by 4.23%, precision by 4.26%, recall by 4.27%, and F1-score by 4.28%. Performance drops significantly after removing the adaptive agg-modality fusion module and the contrastive learning strategy. The procedure indicates that these two components are complementary in the CLAAF model. The adaptive agg-modality fusion module captures complex inter-modal interaction information through multi-level feature fusion, while the contrastive learning strategy enhances feature consistency by improving modality alignment. They enhance the model’s capacity to process multimodal data with greater accuracy and robustness.

### Visualization analysis

**Fig 2:**
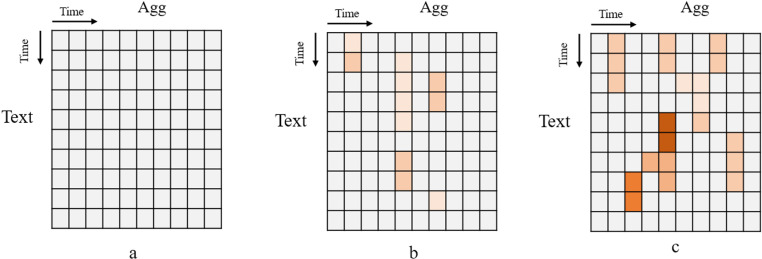
Feature representations at different aggregation layers.

**Fig 3:**
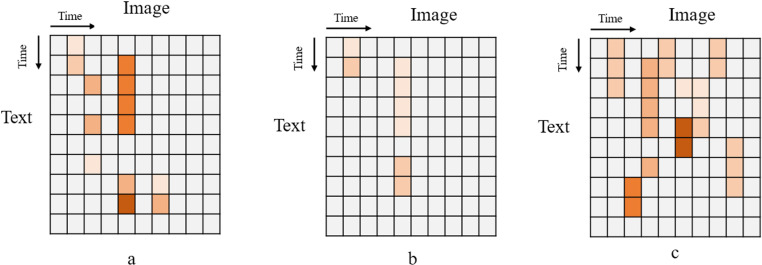
Feature representations at cross-modal interaction layers.

Visualization analysis was conducted to validate the effectiveness of the adaptive agg-modality fusion module. This analysis showcases the feature representations at different aggregation layers (Agg) and cross-modal interaction layers (Text to Image) within the adaptive agg-modality fusion module. The experimental results are illustrated in Figs 2 and 3.

Fig 2 illustrates the changes in feature representations across different aggregation layers. It explains how each layer adds to and improves the consistency of the features. The color grids in the figure represent multimodal features, with darker colors indicating more vital feature consistency. There are no color grids in the first aggregation layer (a), which means that the feature representations of text and images have yet to be fused. Because the features of the two modalities are different in the initial state. As the aggregation layer deepens, the model gradually gathers and optimizes the feature representations of the two modalities. The features start to group together in the intermediate aggregation layer (b). There is more mutual information between the different modalities. Finally, the feature representations become denser in the deep aggregation layer (c), indicating that the model successfully establishes deep interactions. Besides, some light-color grids in (b) are missing in (c) because the model removes unnecessary information using contrastive learning. Meanwhile, it keeps and strengthens essential features. As a result, the text and image are more consistent in the shared space.

Fig 3 shows how the attention mechanism in the cross-modal interaction layer gradually improves the connection between text and images. The color grids in the figure represent multimodal features. The more clustered the grids are, the stronger the feature association is. The model’s attention-weight connections are sparse in the initial interaction layer (a). Although many feature representations exist, most of them focus on a single data type and don’t work together. The interaction of text and image features needs to be clarified. With model training, the weights for cross-modal attention increase in the middle layer (b). The attention mechanism discards the unique unimodal features that appear in (a), and retains only the tightly interacting multimodal features. Finally, the attention weights become dense in the deep interaction layer (c) and exhibit high consistency. The model establishes a solid semantic connection between the text and image modalities. The cross-modal interaction layer uses text features to guide the expression of image features, ensuring that the image modality is semantically consistent with the text information, thereby enhancing the feature alignment effect. This layer-by-layer progressive cross-modal attention mechanism ensures the model can efficiently exchange and fuse information within multimodal features.

### Loss function analysis

**Fig 4:**
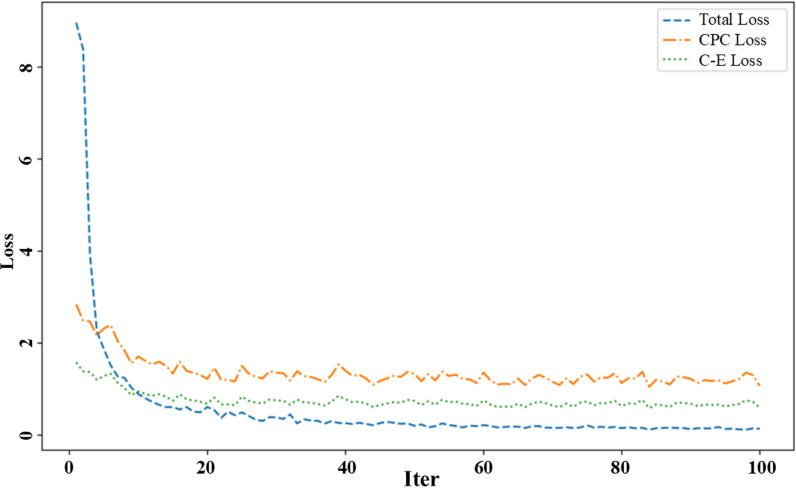
Loss Function analysis.

This study employs two loss functions to train the model: cross-entropy loss and contrastive learning loss. The classification task uses cross-entropy loss to quantify the disparity between the model’s predictions and the actual labels. Minimizing cross-entropy loss allows the model to fine-tune parameters for more accurate predictions of the information category. Contrastive learning loss is applied to handle data from image and text modalities. By comparing positive and negative samples within the same batch, the model enhances its ability to merge features from various modalities, thereby improving the accuracy of information classification. The total loss function is derived by integrating cross-entropy and contrastive learning losses. The trends of these different loss functions during the training process are shown in Fig 4.

As shown in Fig 4, the total loss decreases rapidly during the early stages of training, dropping from an initial high value of approximately 8.5 to a lower value near 0.5 and then gradually stabilizing. The model constantly adjusts its parameters to minimize cross-entropy and contrastive learning loss and boost overall performance. The contrastive learning loss decreases rapidly at the beginning of training, from an initial value of about 2.5 to a lower level close to 1, and then remains stable. The model continually improves its ability to align features from various modalities and increases stability over time. The classification loss steadily declines throughout the training process, from an initial value of around 2 to a lower level near 0.5. The model consistently enhances its classification ability, resulting in higher prediction accuracy.

By jointly using cross-entropy and contrastive learning loss, the CLAAF model can simultaneously focus on classification accuracy and the effectiveness of modality alignment in the training process. Strengthening the model’s capacity to accurately understand the complex interactions between different modalities while processing multimodal data can improve classification outcomes.

## Conclusion and future work

### Conclusion

Multimodal fake information detection is essential for maintaining information authenticity and credibility across various platforms. This paper proposes a novel model utilizing Contrastive Learning and Adaptive Alignment Fusion (CLAAF), notably improving the precision and effectiveness of detecting multimodal fake information. The conclusions are as follows.

Firstly, the proposed contrastive learning strategy improves modality consistency and feature representation quality. By implementing an alignment first and fusion later approach through contrastive learning and text-guided image modalities, the CLAAF model preserves aligned features while eliminating redundant information. Experimental results demonstrate that models utilizing this strategy achieve a 2.85% increase in accuracy compared to those without it, underscoring its critical role in enhancing classification performance.

Secondly, introducing an agg-modality fusion module enables adaptive learning and comprehensive integration of multimodal information. This module performs layer-by-layer aggregation and alignment of features, where text features guide and optimize image feature representations, thereby improving the model’s understanding and processing capabilities. Experiments show that incorporating the multi-scale fusion module leads to a 2.46% improvement in accuracy, highlighting its effectiveness in boosting detection performance.

Thirdly, this study constructs a more extensive, diverse, and authentic multimodal fake information dataset to address the limitations of existing datasets. The dataset comprises text and image pairs verified by authoritative platforms, ensuring high authenticity and representativeness. This dataset facilitates practical model training and evaluation and exhibits the experimental outcomes’ reliability and generalizability, demonstrating the CLAAF model’s applicability across various real-world scenarios.

In summary, the proposed CLAAF model effectively leverages contrastive learning and adaptive agg-modality fusion module to advance the detection of multimodal fake information overtly. The model outperforms baseline methods across all evaluation metrics, showcasing its superiority in complex information environments. Additionally, the newly developed dataset provides valuable resources for future research, confirming the study’s robustness and the practical application of its findings.

### Limitations and future work

Although CLAAF has demonstrated superior performance in multimodal fake information detection, it still has some limitations for further optimization. On the one hand, the model may face challenges when dealing with highly complex or extremely imbalanced datasets. In some practical situations, the proportion of fake information may be less than that of factual information, making enhancing the model’s performance challenging. Future research should explore more advanced sampling techniques or adjustments to the loss function to improve the model’s performance. On the other hand, while the CLAAF model has shown good computational efficiency in experiments, its complexity may still need further optimization for application in low computing power environments. Future research should focus on simplifying and optimizing the model structure to reduce computational complexity. In addition, we plan to collect information from other social media platforms to enrich our dataset, such as Twitter. This can further validate our findings and ensure the robustness of our model.

## Supporting information

S1 File Real information of the text part of our constructed dataset. (CSV)

S2 File False information. (CSV)
